# Consumption of Dental Treatment in Patients with Inflammatory Bowel Disease, a Register Study

**DOI:** 10.1371/journal.pone.0134001

**Published:** 2015-08-12

**Authors:** Annsofi Johannsen, Michael C. Fored, Jan Håkansson, Anders Ekbom, Anders Gustafsson

**Affiliations:** 1 Department of Dental Medicine, Division of Periodontology, Karolinska Institutet, Huddinge, Sweden; 2 Department of Medicine Solna, Clinical Epidemiology Unit T2, Karolinska Institutet, Stockholm, Sweden; 3 Department of Periodontology, Institute of Odontology, The Sahlgrenska Academy at University of Gothenburg, Gothenburg, Sweden; CWRU/UH Digestive Health Institute, UNITED STATES

## Abstract

**Objective:**

The aim of this study was to compare the consumption of dental treatment among patients with Crohn´s disease (CD) or ulcerative colitis (UC) compared to age and gender matched control groups.

**Design:**

The study group comprised 2085 patients with CD and 3161 with UC from the Uppsala-Örebro region and from the Stockholm region. The patients in the cohort were diagnosed between 1960 and 1989. Patients up to 70 years of age were included in the study. The two patients groups were compared to age- and gender-matched, randomly selected control groups from the same geographic area comprising a corresponding number of participants.

**Results:**

CD patients had significantly higher total number of procedures registered (p < 0.000). The difference was most pronounced for removable dentures (+65%), fillings in front teeth (+52%) and endodontic treatment (+46%) when Crohn’s patients were compared to controls (p<0.001). The corresponding figures for UC patients were also a significantly higher total number of procedures (p < 0.005), more clinical examinations (p<0.000), fillings in canines and incisors (p < 0.001) and fillings in bicuspids and molars (p < 0.000).

**Conclusion:**

This study demonstrate that CD and UC individuals use more dental treatment compared to an age-gender matched control group, and more caries-related treatments. The difference was most pronounced for restorative treatment in patients with Crohn’s.

## Introduction

Inflammatory bowel diseases (IBD) consist of two chronic recurrent inflammatory disorders of the gastrointestinal tract: Crohn’s disease (CD) and Ulcerative colitis (UC). CD affects any part of gastrointestinal tract whereas UC affects only the large bowel [1]. The exact etiology of IBD is not known, however several studies have shown that genetics in pathogenesis and environmental factors is involved. [2].

The diagnoses of CD and UC is based on clinical signs, endoscopy, histology, radiographic and/or biochemical investigation. [3]. Signs and symptoms of these diseases are abdominal pains, fatigue, weight loss, diarrhea, poor appetite and rectal bleeding and shows episodes with disease activity, such as exacerbation, asymptomatic intervals, and remissions. [4].

A possible association between IBD and oral disease have been investigated earlier but the findings are not entirely conclusive. Previous studies have found a higher prevalence of caries in CD patients in comparison to healthy individuals. [5, 6]. Brito et al. reported significantly higher of DMF-T (decayed, missed, filled teeth) in CD and UC patients compared to a control group. [7] A questionnaire study revealed that patients with CD reported significantly more mouth-related problems and a greater need for dental treatment compared to a control group. [8]. Concerning periodontits, which is a multi-factorial chronic inflammatory disease and characterized by destruction of gingival connective tissue and can lead to tooth loss. [9]. Periodontitis share pathogenic features with CD, both these diseases seem to have an overreactive inflammatory response with an increased production of free radicals. [10, 11]. Another factor is cigarette smoking, which is strongly associated with periodontitis and CD [12, 13] while being indicated as protective against UC. [14]. A higher prevalence of periodontitis in patients with IBD have been shown in a number of studies. Flemmig et al. showed that patients with IBD, without specifying which disease, had more sites with attachment loss compared to the general US population (p<0.01) but the the attachment loss was generally less severe (p<0.01). [15] Grössner-Schreiber et al., showed that patients with IBD had more periodontal pockets and more clinical attachment loss. [16] Brito et al. showed that both patients with CD and patients with UC had a higher prevalence of periodontitis. [7] A recent Jordanian study showed that both CD and UC had a signficanly higher prevalence of perioodntitis (odds ratio 4.9 and 7.0 respectively. [17]. A prospective study from USA showed that IBD was associated with periodontitis. [18].

Both CD and UC are characterised by a hyper reactive inflammatory response but seem to have different aetiology and pathogenesis and should therefore be assessed separately [19].

A new dental insurance system was incorporated in the general oral health insurance system in Sweden from 2008, this has made it possible to follow the pattern of dental care consumption for different groups of patients. The present study will provide information about the total amount of dental treatment that patients with CD and UC have consumed and it will also provide important information about the kind of treatment needed in the two groups. Earlier studies that had investigated the prevalence of caries and periodontitis are rather few and with relative small sized groups and no study has investigated dental consumption in IBD patients.

Therefore, the object of this study to test the hypothesis that patients with CD and UC have a higher consumption of dental treatment compared to a control group.

## Materials and Methods

### Patients

Ethical approval for this study was obtained from the Karolinska Institutet Ethical Research Board (ref. 2010/496-31/3). No individual consent was obtained or required in this register based study. All patient records/information was anonymized and de-identified prior to analysis.

The study group comprised an established cohort of patients with Inflammatory Bowel disease (Ulcerative colitis (UC) and Crohn´s disease (CD)) from the Uppsala-Örebro region and from the Stockholm region. The cohort consisted of 2085 patients diagnosed with UC and 3161 patients with CD. [20]. The patients in the cohort were diagnosed between 1960 and 1989. Patients up to 70 years of age were included in the study.

Each patient group was compared to an age-, gender–and geographically matched, randomly selected control group comprising a corresponding number of controls.

### The dental register

The Swedish Social Insurance Agency registers all dental treatment performed by dentist or dental hygienist, connected to the national dental insurance system, approximately 99% of all dental practitioners. All dental treatment delivered to the participating patients and controls for 12 months, starting any time during 2009 were recorded.

The dental treatment was divided into 12 different categories; clinical examinations, radiographic examinations, specific caries treatment (including dietary counseling and treatment with various fluoride preparations), periodontal treatment (including scaling and root planning and periodontal surgery), surgery (including extractions and more extensive dentoalveolar surgery), implant installations, endodontic treatment, fillings in canines and incisors, fillings in bicuspids and cuspids, fixed dental prostheses, removable prostheses, implant supported prostheses and the total number of procedures.

### Statistical analyses

Analyses of the data were performed using the software package PASW Statistics 18 (PASW Inc., Chicago, IL, USA). The data is skewed since most participants did not receive many of the various proceedings. The distributions were tested with the Kolmogorov-Smirnov and Shapiro-Wilks tests, which both showed a statistically significant deviation from normality (p<0.000). Thus, the significance of differences between patients (CD, UC) and controls, and the difference between men and women were calculated with Mann-Whiney U-test. However, in order to illustrate the differences between the groups we opted to present the results as mean and standard deviation. The correlations between disease duration and dental treatments were calculated with Spearman´s rank correlation coefficient.

## Results

The demographic data over the patients and controls are presented in Table 1.

**Table 1:** Demografic data for 3161 patients with Crohn’s disease and 2085 patients with Ulcerative colitis, and their gender and age matched controls.

Variable	Crohn’s disease	Controls	Ulcerative colitis	Controls
Age	53.1 (10.3)	53.1 (10.3)	57.0 (8.2)	57.0 (8.2)
Gender F/M (%)	51.7/48.3	51.7/48.3	46.8/53.2	46.8/53.2
Age at diagnosis	26.0 (9.9)		23.0 (8.2)	
Disease duration	27.1 (11.1)		34.1 (8.0)	

Regarding the dental treatment, CD patients had significantly higher total number of procedures registered (p < 0.000). The difference was most pronounced for removable dentures (+65%), fillings in front teeth (+52%) and endodontic treatment (+46%) when Crohn’s patients were compared to controls (p<0.001) (Table 2). The corresponding figures for UC patients were also a significantly higher total number of procedures (p < 0.005), more clinical examinations (p = 0.005), fillings in canines and incisors (p < 0.001) and fillings in bicuspids and molars (p < 0.000) (Table 3). The distribution of dental treatment was very skewed, most participants in both patient and control groups having had none or one treatment procedure in each category (S1 Fig).

**Table 2:** Dental treatment in 3161 patients with Crohn’s and in 3161 age, gender and geographically mached controls. Mean number of procedures ±standard deviation. Significance of differences between patients and controls calculated with Mann-Whitney U test.

	All patients with Crohn’s and corresponding controls			
Number of procedures within the following categories	Crohn’s	Controls	Ratio P/C	p-value
Clinical examination	2.13 ± 1.97	1.89 ± 1.78	1.13	0.000
Radiographic examination	0.49 ± 0.98	0.42 ± 0.94	1.17	0.004
Specific caries treatment	0.44 ± 0.90	0.43 ± 0.89	1.02	0.731
Periodontal treatment	1.12 ± 1.78	1.05 ± 1.68	1.07	0.022
Surgery	0.29 ± 1.13	0.22 ± 0.97	1.32	0.000
Implant installations	0.04 ± 0.38	0.06 ± 0.57	0.67	0.724
Endodontic treatment	0.19 ± 0.68	0.13 ± 0.53	1.46	0.000
Fillings in canines and incisors	0.35 ± 0.96	0.23 ± 0.76	1.52	0.000
Fillings in bicuspids and cuspids	1.19 ± 1.81	0.91 ± 1.55	1.31	0.000
Fixed dental prostheses	0.42 ± 1.68	0.32 ± 1.40	1.31	0.000
Removable prostheses	0.13 ± 1.19	0.08 ± 0.75	1.62	0.089
Implant supported prostheses	0.04 ± 0.40	0.03 ± 0.40	1.33	0.303
Total number of procedures	6.86 ± 7.13	5.79 ± 6.21	1.18	0.000

**Table 3:** Dental treatment in 2085 patients with Ulcerative colitis and in 2085 age, gender and geographically mached controls. Mean number of procedures ±standard deviation. Significance of differences between patients and controls calculated with Mann-Whitney U test.

	All patients with ulcerative colitis and corresponding controls			
Number of procedures within the following categories	Colitis	Controls	Ratio P/C	p-value
Clinical examination	2.04	1.88	1.08	0.000
Radiographic examination	0.45	0.45	1.00	0.497
Specific caries treatment	0.47	0.43	1.09	0.123
Periodontal treatment	1.13	1.13	1.00	0.009
Surgery	0.24	0.21	1.14	0.914
Implant installations	0.05	0.05	1.00	0.916
Endodontic treatment	0.17	0.15	1.13	0.240
Oral physiology	0.03	0.02	1.50	0.302
Fillings in canines and incisors	0.32	0.24	1.33	0.000
Fillings in bicuspids and molars	1.20	0.95	1.26	**0.000**
Fixed dental prostheses	0.38	0.42	0.90	0.497
Removable prostheses	0.07	0.05	1.40	0.502
Implant supported prostheses	0.03	0.03	1.00	0.285
Total number of procedures	6.57	6.01	1.09	0.000

Women in general had more dental treatment compared to men (p = 0.000). The differences were significant regarding clinical examinations (p<0.001), radiographic examinations (p<0.01) and caries treatment (p<0.001). This tendency was the same in all 4 groups, CD patients, UC patients and the two control groups.

There were weak but signficant correlations between the total number of procedures and disease duration after compensation for age both CD (r = 0.035, p = 0.047) and UC (r = 0.047, p = 0.033).

## Discussion

In this population-based cohort study, we found that both UC and CD patients had significantly more dental procedures during a year compared to a randomly selected age- and gender-matched groups from the same geographic areas. This difference was most pronounced when the Crohn’s patients (CD) were compared to their controls. Due to the relatively large number of comparisons should differences in specific treatment procedures be interpreted with caution. Anyhow, the biggest difference was seen in procedures related dental caries or tooth loss. Treatment with removable dentures was 62% more common among the Crohn patients compared to the controls. Endodontic treatment was 46% more common and fillings in canines and incisors were 52% more common. This indicates the Crohn’s patients had higher caries prevalence.

The differences were less pronounced in the Ulcerative colitis (UC) group but they also had more fillings done.

Increased caries prevalence in UC and CD patients has been shown in earlier clinical studies who reported a higher DMT-T and DMT-S (decayed, missed filled surfaces) [6, 7]. Diagnosed with CD and UC many times leads to a change in the individual’s diet *e*.*g*. with increased meal frequency and high intake of carbohydrates which can have a negative impact for the patient’s oral health with increased caries risk. In a recent prospective cohort study from the European Cronh´s and Colitis Organization (ECCO), Epidemiological Committee (EpiCom), who represented 31 European countries, investigate the impact of environmental factors in CD and UC, and compare Eastern and Western IBD patients. [21]. A rapid increase incidence in these diseases during the last decades in Eastern Europe have been observed, which could not only be explained by genetic susceptibility. The study found among other things, that sugar intake was significantly higher in CD and UC prior to diagnose in Eastern European. This results support the theory of Westernization of lifestyle having a main role in the recent incidence increase in IBD [21]. High sugar intake in UC and CD patients has also been reported from other studies [6, 17, 22], showing that high sugar intake was associated with CD but not with UC which could be an explanation for the differences in treatment found in our study. [23]

Dietary habits play an important role in shaping the microbiota of the human gut (Wu et al. 2011) and a “Westernized” diet hypothesized to alter the intestinal microbiota towards a composition which increase the risk of IBD. [24].

From a gender perspective, women in both patient groups in the present study consumed slightly more dental care, albeit the difference only reached significance regarding clinical examinations. However, this difference was not associated with the disease, women in the control groups showed a similar tendency.

A major strength of this study is its large number of individuals in conjunction with the independent identification of cases through national, Swedish Social Insurance Agency.

The advantages of this kind of register study is that provide a comprehensive overview and estimation of the need for dental treatment among patients with UC and CD, especially when this patients need to change *e*.*g*. the diets and are more prone to inflammation, which may have an influence on the oral health. In other words, it can identify various risk factors for oral diseases, such as caries and periodontal diseases.

The present study showed that this patient’s group visit more frequent to dental services, which indicate that they had a more need for dental treatment, which are in line with a questionnaire study in CD patients. [8]. It need to be mentioned that the improved conditions in dental care among people in the Scandinavian countries imply that the number of radical treatment, such as tooth extractions and extensive prosthodontic treatments, has declined, while the number of people who visit dental services for preventive reasons has increased. It would be interesting to investigate other European countries in that purpose. Most patients and controls had very little dental treatment done but the number of participants having more than one treatment procedure were higher in the patients groups.

This study has some limitations that need to be addressed. We have no data on the occurrence of tobacco use but a previous study from our group showed that there were significantly more smokers and former smokers in a group of patients with Crohn´s compared to matched controls. [8] Smoking is risk factor for CD, but in UC smoking appears to have a protective effect. [25]. The contradictory effects of smoking in IBD have been discussed that smoking has a more organ-specific, rather than a disease-specific effect. [26]. In the present study, there was no significantly difference regarding number of procedures of periodontal treatment among the groups. The result was somewhat surprising, because smoking is a known important environmental risk factor for periodontitis and other chronic inflammatory disease. [27]. In our present study the most pronounced differences were seen in treatment procedures that is related to caries. There are studies indicating an association between caries and tobacco smoking but the relationship is not very clear. [28]. Furthermore, clinical studies have shown that patients with CD have more periodontitis than controls [8, 18, 29] and significantly more gingival inflammation than the controls (Szymanska, unpublished observations). This inconsistency needs to be further investigated. For example, it is reasonable to believe that medication used to treat IBD also have an effect on the periodontium but this is not yet entirely clarified. Another possible weakness is that we have not controlled for the occurrence of IBD in the control group. It is reasonable to believe that the prevalence of IBD is the same in the control group as in the Swedish population, i.e. x%, which would not have a significant influence on the results. Furthermore, the results could be influenced by other systemic diseases that could influence oral health, primarily diabetes. However, considering the relatively large number of participants we expect these disease to be evenly spread in all three groups.

We have not related the dental treatment to IBD disease activity, which could influence both the need for dental treatment and the ability to actually seek treatment. Vavricka et al. found no clear association between clinical activity and periodontitis, except for perianal manifestations in CD patients. [18] Clarifying the influence of disease activity, smoking and medication would require prospective studies.

## Conclusions

In conclusion, this study demonstrates that CD and UC individuals use more dental treatment compared to an age-gender matched control group, and more caries-related treatments. The difference was most pronounced for reparative treatment in patients with Crohn’s.

## Supporting Information

S1 Fig Showing the distribution of the total number of dental treatment procedures in patients with Crohns and ulcerative colitis, and respective controls. (PDF)

S1 Dataset Individual level data behind the statistics reported in the manuscript. (XLS)
