# Supplementary material for: Consumption of Dental Treatment in Patients with Inflammatory Bowel Disease, a Register Study
Source: PLoS One. 2015 Aug 12;10(8):e0134001. doi: 10.1371/journal.pone.0134001 (PMC4534207; doi:10.1371/journal.pone.0134001)
Supplement: S1 Fig — (PDF) [file pone.0134001.s001.pdf]

Supporting information

1B

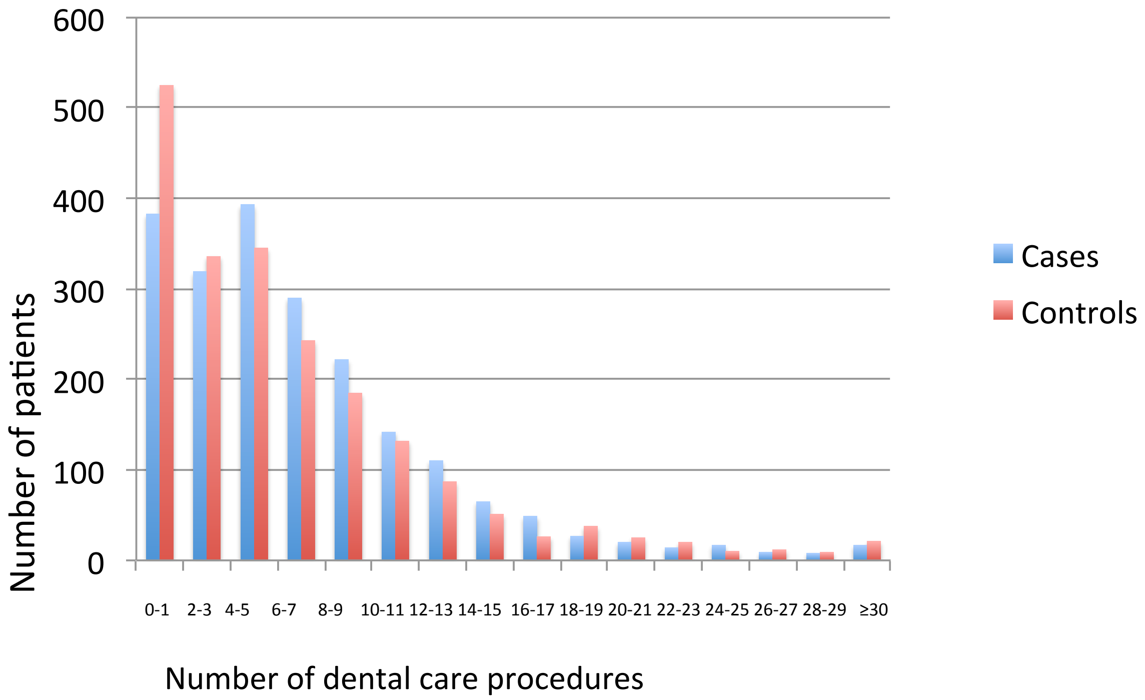

1A

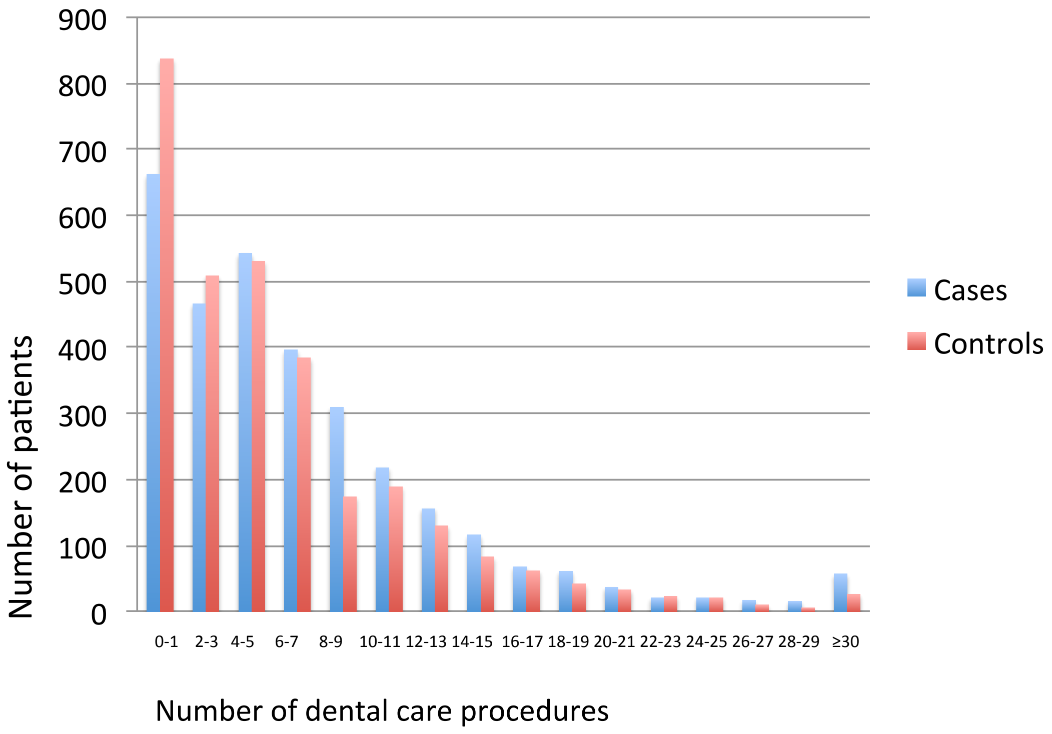

Histogram showing the total number of dental care procedures during one year (2009-2010) for 2085 patients with Crohn´s disease (1A) and 3161 patients with Ulcerative colitis (1B), and their corresponding controls.
